# Left atrial stiffness in women with ischemia and no obstructive coronary artery disease: Novel insight from left atrial feature tracking

**DOI:** 10.1002/clc.23395

**Published:** 2020-05-27

**Authors:** Sauyeh K. Zamani, Thomas Jake Samuel, Janet Wei, Louise E. J. Thomson, Balaji Tamarappoo, Behzad Sharif, C. Noel Bairey Merz, Michael D. Nelson

**Affiliations:** ^1^ Department of Kinesiology University of Texas Arlington Texas USA; ^2^ Barbra Streisand Women's Heart Center Smidt Heart Institute, Cedars‐Sinai Medical Center Los Angeles California USA; ^3^ Biomedical Imaging Research Institute Cedars‐Sinai Medical Center Los Angeles California USA

**Keywords:** feature tracking, heart failure with preserved ejection fraction, ischemia with no obstructive coronary artery disease, left atrial strain

## Abstract

**Background:**

Women with signs and symptoms of ischemia and no obstructive coronary artery disease (INOCA) are at risk of heart failure with preserved ejection fraction (HFpEF); however, the mechanism for HFpEF progression remains unclear. Studies in INOCA have largely focused on left ventricular function. The left atrium serves an important role in maintaining transmitral flow, and is impaired in HFpEF; however, it remains unclear if left atrial function is impaired in INOCA.

**Hypothesis:**

Left atrial function is progressively worse in INOCA and HFpEF compared to controls.

**Methods:**

We compared 39 reference control subjects to 64 women with INOCA and 22 subjects with HFpEF. Left atrial strain was assessed by feature tracking using magnetic resonance cine images.

**Results:**

Peak left atrial strain was reduced in HFpEF compared to controls (22.9 ± 4.8% vs 25.9 ± 3.2%, *P* < .01), but similar in INOCA (24.8 ± 4.5%) compared to HFpEF and controls (*P* = .18). However, left ventricular end‐diastolic pressure (LVEDP) was elevated in 33% of INOCA participants, suggesting that left atrial stiffness (LVEDP/LA strain) is elevated in a large portion of women with INOCA.

**Conclusions:**

Taken together, we interpret these data to support our working hypothesis that INOCA is a pre‐HFpEF state, with left atrial stiffness preceding overt left atrial dysfunction; representing a putative therapeutic target to prevent HFpEF progression in this at‐risk population.

## 1 INTRODUCTION

Ischemic heart disease is the leading cause of death in women, with annual mortality rates exceeding all forms of cancer combined.^1^ Work from the NHLBI‐Sponsored Women's Ischemia Syndrome Evaluation (WISE) study suggests that most women presenting with signs and symptoms of ischemia have no obstructive coronary artery disease (INOCA),^2^ and that these women are at increased risk of developing heart failure^3^; confirmed to be almost exclusively heart failure with preserved ejection fraction (HFpEF).^4^ However, despite our increased understanding, the pathophysiologic mechanism(s) driving heart failure progression in INOCA remains incompletely understood.

Consistent with the HFpEF phenotype, our group has shown that women with INOCA often have left ventricular diastolic dysfunction^5^, ^6^, ^7^, ^8^; however, these investigations have focused almost exclusively on left ventricular relaxation. The left atrium also contributes in generating the transmitral pressure gradient needed to fill the ventricle, and multiple investigations have shown that left atrial function provides important insight into the adaptive changes that contribute to ventricular filling.^9^, ^10^, ^11^, ^12^, ^13^, ^14^, ^15^, ^16^, ^17^ To the best of our knowledge, no study has evaluated left atrial function in women with INOCA. Given that left atrial function is often impaired in HFpEF,^16^, ^18^, ^19^, ^20^, ^21^ we hypothesized that left atrial function would also be impaired in INOCA.

## 2 METHODS

### 2.1 Study population

To test our specific hypothesis, left atrial function was assessed in 40 reference control women, 64 women with INOCA, and 23 women and men with HFpEF, enrolled in the WISE‐HFpEF study (NCT02582021, enrolled in October2015‐Feb 2020), or recruited as part of our reference control registry. Women with INOCA presented with persistent signs and symptoms of ischemia but had <50% coronary artery stenosis confirmed by angiography. As part of the larger trial design, left ventricular end‐diastolic pressure (LVEDP) was measured by a pressure catheter inserted through a peripheral artery and placed into the lumen of the left ventricle. Women and men with stable chronic HFpEF were recruited from the outpatient setting if they met modified European Society of Cardiology criteria^22^ that included: symptoms of heart failure, left ventricular ejection fraction ≥45%, structural evidence of cardiovascular abnormalities (evidence of abnormal filling or relaxation, left atrial enlargement, or left ventricular hypertrophy documented by echocardiogram), and evidence of elevated left ventricular filling pressure (LVEDP or pulmonary capillary wedge pressure at rest >15 mmHg and/or with exercise ≥25 mmHg, b‐type natriuretic peptide >100 pg/mL, or current use of diuretic). HFpEF subjects were excluded if they had atrial fibrillation at time of imaging, significant valvular heart disease, significant chronic pulmonary disease, or known history of hypertrophic or infiltrative cardiomyopathy or constrictive pericarditis. Obstructive coronary artery disease was ruled out in HFpEF subjects using cardiac‐computed tomographic angiography.^23^ Reference control women did not have any symptoms, risk factors for, or evidence of ischemic heart disease, confirmed by a standardized 12‐lead treadmill stress test.^24^ All study subjects gave written informed consent before undergoing evaluation and the study protocol was approved by the Institutional Review Board at Cedars‐Sinai Medical Center.

### 2.2 Cardiac magnetic resonance imaging

In the majority of subjects, cardiac magnetic resonance imaging was performed on a 3.0T scanner (Siemens Healthineers, Erlangen, Germany); however, a subset of participants (n = 27) underwent cardiac magnetic resonance imaging on a 1.5T scanner (Siemens Healthineers, Erlangen, Germany). In all cases, images were electrocardiogram‐gated and a phase‐array surface coil (CP Body Array Flex; Siemens Healthineers) was used. Long‐axis views (ie, 4‐, 3‐, and 2‐chamber) along with a series of short‐axis cine images spanning the entire left ventricle were collected (steady‐statefree‐precession pulse sequence) for assessment of left ventricular and left atrial morphology and function.

Left ventricular mass and volumes were assessed using the method of disks, from a series of short‐axis cine images spanning the entire left ventricle, as previously described.^25^ Briefly, using the commercially available software (CVI^42^ version 5.6.8; Circle Cardiovascular Imaging Inc, Calgary, AB, Canada or CAAS MRV, Pie Medical Imaging, B.V., Netherlands), endocardial and epicardial borders were drawn manually at end‐diastole and end‐systole on all short‐axis slices that included ventricular mass and volume, with care taken to avoid including blood volume from the left ventricular outflow tract. Left ventricular mass and volumes were reported as absolute and indexed to body surface area.^26^ Left ventricular ejection fraction was calculated as stroke volume divided by end‐diastolic volume, expressed as a percentage.

To evaluate left atrial strain, the horizontal and vertical long‐axis cine images (25 cardiac phases) were analyzed by feature tracking using the CVI^42^ software (version 5.6.8; Circle Cardiovascular Imaging Inc, Calgary, AB, Canada). A single experienced observer (S. K. Z.), blinded to the clinical status of each subject, manually delineated the endocardial and epicardial borders of the left atrium, on a single cardiac phase at left ventricular end‐systole (just prior to mitral valve opening), before applying the feature tracking algorithm across the remainder of cardiac phases. All strain measurements were performed in duplicate; however, if discrepancies between repeat measurements were identified, a third attempt was performed. Data were only included if it satisfied internal standards, primarily based on image and tracking quality. Insufficient tracking was defined as a visually apparent deviation of the contours from the endocardial and/or epicardial borders. In such a case, the contours were manually corrected and the feature‐tracking algorithm was reapplied. A second experienced observer (M. D. N.), also blinded to the clinical status of the subject, reviewed and confirmed all included data. Reported strain measurements represent an average of each measurement trial for each subject. Data were excluded if both reviewers were not satisfied with the tracking quality. Left atrial function was characterized by three distinct phases: reservoir, when the left atrium passively receives blood from the pulmonary circulation; conduit, when blood flows passively from the atrium to the ventricle along the transmitral pressure gradient; and booster, when the left atrium contracts, transferring blood into the ventricle. Intraobserver variability for reservoir, conduit, and booster strain, reported as a coefficient of variation, are 2%, 4%, and <1%, respectively. Left atrial stiffness index was defined by the ratio between LVEDP and peak reservoir strain, as previously reported.^27^, ^28^, ^29^, ^30^, ^31^ To define the threshold of normal, all control subjects were assumed to have a LVEDP of 12 mmHg (the upper limit of normal), with the threshold for abnormal left atrial stiffness set two SD above the calculated mean.

Left atrial volume was measured using the same horizontal and vertical long‐axis images, along with the left ventricular outflow tract view (ie, 3‐chamber), as previously described,^32^ at three specific time points: end of ventricular systole (immediately prior to mitral valve opening) to assess left atrial reservoir volume, early ventricular diastole (immediately prior the atrial contraction) to assess left atrial conduit volume, and at late ventricular diastole (immediately following mitral‐valve closure) to determine left atrial booster volume. Left atrial ejection fraction was calculated as the difference between reservoir and booster volume divided by reservoir volume, expressed as a percentage.

### 2.3 Statistical methods

Data were analyzed using IBM SPSS Statistics 24 (version 13.0). All data are reported as mean ± SD, unless otherwise specified. Group comparisons were made using either one‐way analysis of variance or Kruskal‐Wallis tests and chi‐square or Fisher exact tests for categorical variables. Analysis of covariance was also performed on measures of left atrial function to reduce error associated with between‐group variance in age and body mass index. When necessary, study variables were transformed to approximate normality. When a significant difference was found, post hoc testing for specific group differences was performed using LSD (parametric) or Mann‐Whitney (nonparametric) comparisons. For all tests, two‐sided*P* values of ≤.05 were considered to indicate statistical significance.

## 3 RESULTS

Data analysis for left atrial strain was successful in all but two subjects (2%) who were excluded from the final analysis due to technical challenges associated with image analysis, leaving a total of 39 reference controls, 64 INOCA, and 22 HFpEF subjects. In 3 of the 22 (14%) subjects with HFpEF, conduit and booster strain were not easily discernible, and therefore not included in the analysis.

Subject characteristics are depicted in Table 1. HFpEF subjects were on average older, and had a higher body mass index and body surface area, compared to the other groups. The majority of HFpEF participants were classified as NYHA class II (NYHA class: I, n = 1; II, n = 16; and III, n = 5). Left ventricular end‐diastolic volume and left ventricular mass were similar between groups; however, left ventricular end‐systolic volume was higher in HFpEF and INOCA, compared to controls, resulting in a small but significant difference in left ventricular ejection fraction between groups (Table 1).

**TABLE 1:** Subjects demographics and resting hemodynamics

	Reference control	INOCA	HFpEF	*P*‐value
n	39	64	22	—
Age, years	50 ± 8	55 ± 11^a^	62 ± 11^a^ ^,^ ^b^	<.001
Female	39 (100%)	64 (100%)	18 (82%)	<.001
Caucasian	23 (59%)	55 (86%)	14 (64%)	.004
Body mass index	25.4 ± 3.7	28.1 ± 6.7	30.9 ± 6.8^a^ ^,^ ^b^	.005
Body surface area, m^2^	1.7 ± 0.2	1.7 ± 0.2	1.9 ± 0.2^a^ ^,^ ^b^	.003
Rest hemodynamics				
Heart rate, bpm	62 ± 8	63 ± 8	64 ± 11	.56
Systolic blood pressure, mmHg	121 ± 18	119 ± 14	128 ± 21	.19
Diastolic blood pressure, mmHg	61 ± 11	63 ± 10	68 ± 10	.12
LV end‐diastolic volume, mL	121 ± 24	119 ± 19	130 ± 44	.82
LV end‐systolic volume, mL	38 ± 8	44 ± 10^a^	52 ± 28^a^	.01
LV stroke volume, mL	83 ± 20	75 ± 12	77 ± 18	.08
LV ejection fraction, %	68 ± 5	63 ± 5^a^	61 ± 6^a^	<.001
LV mass, g	76.8 ± 14.4	76.2 ± 12.7	87.9 ± 30.9	.25
Medical history				
Hypertension	0 (0%)	20 (31%)	18 (82%)	<.001
Diabetes	0 (0%)	3 (5%)	4 (18%)	.008
Dyslipidemia	0 (0%)	8 (13%)	5 (23%)	.001
Myocardial infarction	0 (0%)	16 (25%)	0 (0%)	<.001
Medications				
Diuretics	0 (0%)	10 (16%)	16 (73%)	<.001
Angiotensin converting enzyme inhibitors	0 (0%)	19 (30%)	4 (18%)	.002
Angiotensin receptor blockers	0 (0%)	5 (8%)	5 (23%)	.03
Beta blockers	0 (0%)	20 (31%)	13 (59%)	<.001
Calcium‐channel blockers	0 (0%)	22 (34%)	8 (36%)	<.001
Nitrates	0 (0%)	29 (45%)	4 (18%)	<.001

*Note:* Values expressed as mean ± SD or n (%).

Abbreviations: HFpEF, heart failure with preserved ejection fraction; INOCA, ischemia and no obstructive coronary artery disease; LV, left ventricular.

a *P* < .05 vs control.

b *P* < .05 vs INOCA.

**FIGURE 1:**
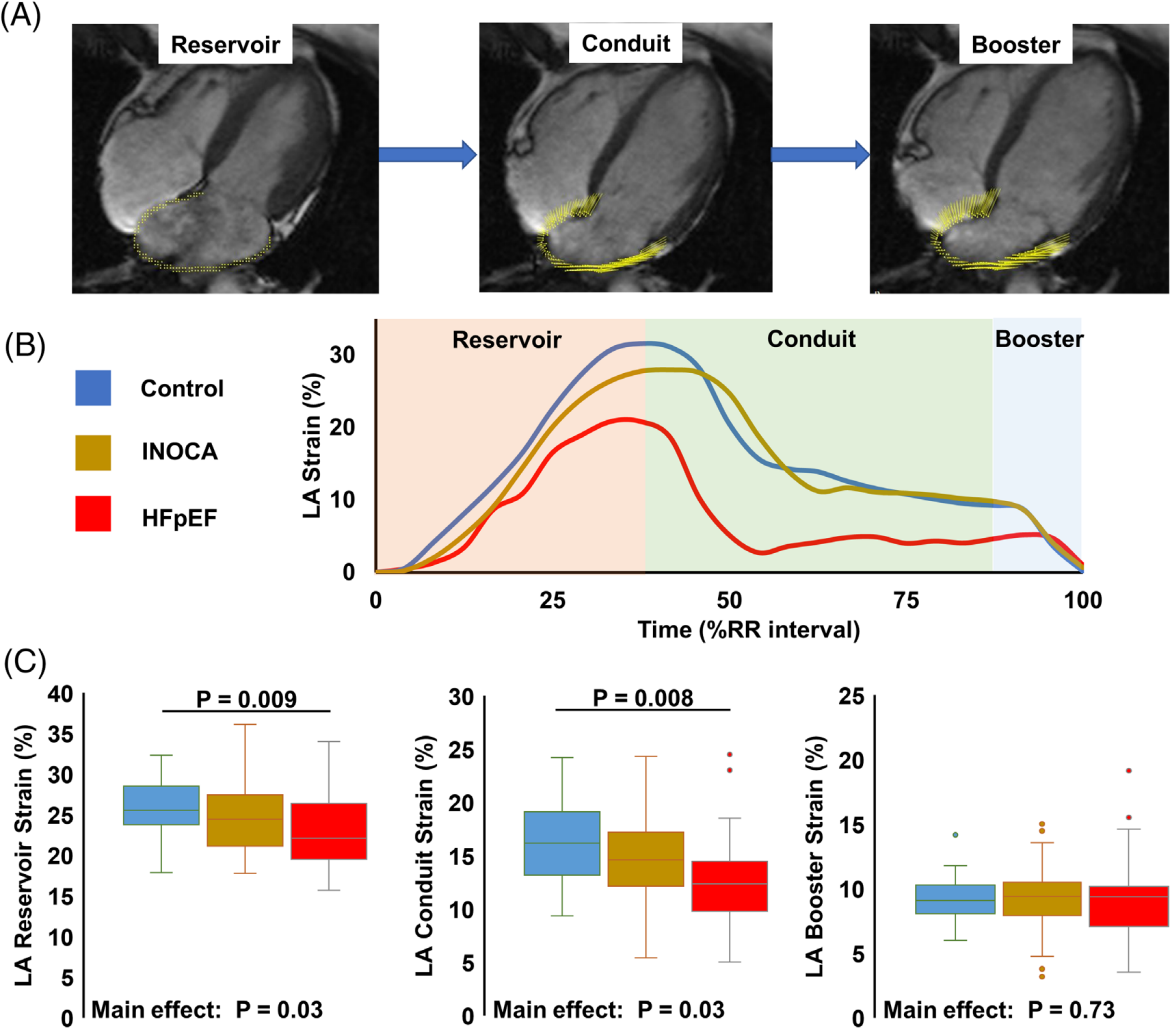
A, High resolution horizontal long‐axis cine image showing left atrial (LA) strain vectors (yellow) derived from the feature tracking algorithm, at each phase of the cardiac cycle: reservoir, conduit, and booster. B, Strain curves from a representative subject from each of the three groups: control (blue), ischemia and no obstructive coronary artery disease (gold), and heart failure with preserved ejection fraction (red). Each LA phase is also highlighted in the background: pink, reservoir; green, conduit; and blue, booster. C, Summary data showing LA reservoir (left) conduit (middle) and booster (right) strain across the heart failure continuum

Consistent with prior reports, left atrial reservoir strain was lower in HFpEF than reference controls, as was left atrial conduit strain (Figure 1). In contrast, left atrial reservoir strain and conduit strain were similar between both controls and INOCA, and HFpEF and INOCA (Figure 1). A similar between‐group pattern was also observed in left atrial volume, with HFpEF having the highest reservoir, conduit, and booster volume compared to controls, with INOCA in the middle (Table 2). After adjusting for age and body mass index, group differences in left atrial strain were eliminated; however, the group differences in left atrial volume persisted (Table 2).

**TABLE 2:** Left atrial morphology and function

	Control	INOCA	HFpEF	*P*‐value	Adjusted *P*‐value
Left atrial volume index, mL/m^2^					
Reservoir	23.7 ± 3.3	30.4 ± 5.5^a^	34.9 ± 10.3^a^ ^,^ ^b^	<.001	<.001
Conduit	18.8 ± 2.8	24.4 ± 4.9^a^	27.1 ± 6.4^a^	<.001	<.001
Booster	13.9 ± 2.4	17.9 ± 4.0^a^	20.9 ± 5.7^a^ ^,^ ^b^	<.001	<.001
Left atrial ejection fraction, %					
	43.2 ± 5.2	41.3 ± 6.0	35.9 ± 8.9^a^ ^,^ ^b^	.005	.03
Left atrial strain, %					
Reservoir	25.9 ± 3.2	24.8 ± 4.5	23.0 ± 4.8^a^	.03	.47
Conduit	16.0 ± 3.8	14.8 ± 4.1	12.8 ± 5.0^a^	.03	.98
Booster	9.1 ± 1.7	9.3 ± 2.2	9.4 ± 3.8	.73	.68

*Note:* Data adjusted for age and body mass index.

Abbreviations: HFpEF, heart failure with preserved ejection fraction; INOCA, ischemia and no obstructive coronary artery disease.

a *P* < .05 vs control.

b *P* < .05 vs INOCA.

**FIGURE 2:**
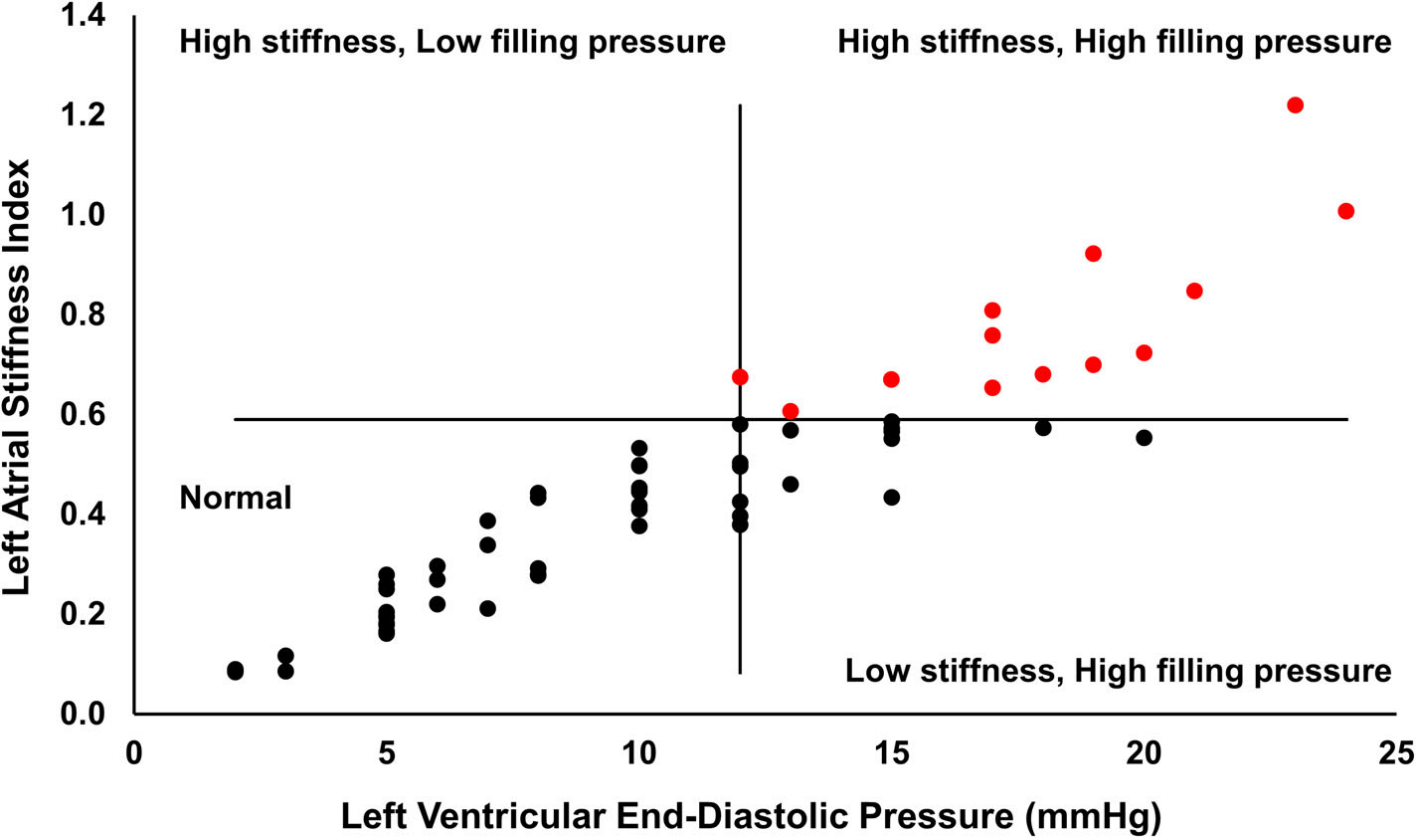
Left atrial stiffness is elevated in a large number of women with ischemia but no obstructive coronary artery disease. Data are divided into four quadrants. Vertical line drawn at 12 mmHg left ventricular end‐diastolic pressure (LVEDP), the upper limit of normal. Horizontal line is drawn at 0.59, which is two standard deviations above the average reference control left atrial stiffness index, estimated by assuming all reference control subjects had an LVEDP of 12 mmHg. Women with ischemia and no obstructive coronary artery disease identified as having elevated left atrial stiffness denoted in red

Remarkably, 33% of INOCA participants had a LVEDP > 12 mmHg, with 19% having an LVEDP ≥ 16 mmHg. Thus, even though left atrial reservoir strain was similar between INOCA and controls, by definition,^28^, ^29^, ^30^, ^31^ a large cohort of INOCA participants had increased left atrial stiffness (Figure 2). Indeed, by assuming all control participants had an LVEDP of 12 mmHg (ie, the upper limit of normal), average left atrial stiffness in the control group was estimated to be 0.47 ± 0.06; placing the threshold for normal at 0.59 (ie, 2 SD above the mean). Based on these conservative estimates, 13 of 64 (20%) INOCA participants had elevated left atrial stiffness (Figure 2).

## 4 DISCUSSION

Multiple investigations have shown that left atrial function provides important insight into the adaptive changes that contribute to ventricular filling.^9^, ^10^, ^11^, ^12^, ^13^, ^14^, ^15^, ^16^, ^17^ This investigation sought to assess left atrial function in INOCA, to expand our pathophysiologic understanding of progression to heart failure in this patient cohort. Our data confirm prior reports^16^, ^18^, ^19^, ^20^, ^21^ showing reduced reservoir strain in HFpEF compared to reference controls. Moreover, while we did not observe a frank reduction in left atrial strain in INOCA compared to controls, left atrial stiffness was elevated in roughly 20% of INOCA participants. Together, these data support left atrial dysfunction as a putative mechanism contributing to HFpEF progression in INOCA.

Previous work by our group has established impaired left ventricular early diastolic strain rate and peak ventricular untwisting rate, as hallmark features of INOCA.^7^, ^8^, ^33^ Here we extend these observations by evaluating left atrial function for the first time in INOCA, and relate these data to HFpEF. Consistent with recent reports, we observed a marked reduction in left atrial reservoir strain in HFpEF compared to reference controls.^18^, ^20^, ^21^, ^34^ In contrast to our original hypothesis; however, we did not observe differences in left atrial strain between INOCA and controls; nor did the differences between HFpEF and controls remain after adjusting for age and body mass index. However, LVEDP was elevated in a third of INOCA participants, suggesting that left atrial stiffness (ie, LVEDP/LA strain) is elevated in a large portion of women with INOCA. While LVEDP was not measured in control participants, we felt it was reasonable to assume that the filling pressures were ≤12 mmHg.^35^ Assuming the upper limit of this cutpoint for all control subjects, we identified 13 of 64 INOCA participants with left atrial stiffness values beyond what could reasonably be explained. This interpretation is further supported by the left atrial volume data, which showed a stepwise increase in reservoir volume from control to INOCA to HFpEF; suggesting that the INOCA group may be operating along the steeper portion of their left atrial compliance curve. Together, these data support our general working hypothesis that INOCA represents a pre‐HFpEF state, whereby subclinical adaptations have not yet manifested into overt remodeling and dysfunction. Whether left atrial stiffness can be reversed in INOCA is beyond the scope of this investigation, but may represent an important therapeutic target. Caution is warranted; however, as simply lowering cardiac filling pressures may have a negative consequence on patients relying on this hemodynamic shift to fill the cardiac chambers. Accordingly, studies addressing the pathophysiologic mechanism(s) driving changes in cardiac hemodynamics in INOCA remain critically important.

## 5 LIMITATIONS

Measuring LVEDP was not clinically indicated in the HFpEF participants at the time of enrollment, nor was it justifiable in the control participants. Surrogate measures of left ventricular filling pressure, like the ratio between early mitral inflow velocity‐to‐early annular tissue velocity, are commonly used to estimate left atrial stiffness.^27^, ^28^, ^29^, ^30^, ^31^ These measures were only available in a subset of participants; however, and therefore not included in the body of the manuscript; despite supporting our overall interpretation (Figure S1). The sample size particularly for the reference control group and HFpEF participants was admittedly low; however, the study was adequately powered to detect differences in our primary endpoint, left atrial reservoir strain. Moreover, the HFpEF participants were older and had a higher body mass index compared to the other two groups. While statistical adjustments were made to account for these group differences, this remains a limitation of this study. Finally, as with other image analysis, the data herein should be interpreted within the confines of both the image modality used to acquire the data (ie, MRI) and analysis software used to determine left atrial strain (ie, feature tracking).

## 6 CONCLUSIONS

The data herein suggest that left atrial stiffness is elevated in a large cohort of women with INOCA. Future investigations are needed to define the mechanism contributing to left atrial stiffness in this patient cohort and to determine if lowering left atrial stiffness can prevent heart failure progression in this at risk population.

## CONFLICT OF INTEREST

C. Noel Bairey Merz: Abbott Diagnostics, Sanofi Vascular, iRhythm.

## Supporting information


**Figure S1** Left atrial stiffness index is elevated in participants with ischemia and no obstructive coronary artery disease (INOCA, gold bar, n = 55) and heart failure with preserved ejection fraction (HFpEF, red bar, n = 15), compared to healthy reference controls (control, blue bar, n = 10). Left atrial stiffness index was estimated by dividing the ratio of early mitral inflow velocity‐to‐early diastolic strain rate (ie, *E*/*e*′_SR_) by left atrial reservoir strain. Early myocardial diastolic strain rate was measured in both the circumferential (panel A) and longitudinal (panel B) directions, by feature tracking of cine images, as described in detail in the body of the manuscript. Mitral inflow velocities were acquired using through plane phase contrast imaging, with the short axis image prescribed at the level of the mitral value leaflet tips. Group comparisons were main using a one‐way analysis of variance, with LSD post hoc comparisons. * indicates *P* < .05.
