# Supplementary material for: Left atrial stiffness in women with ischemia and no obstructive coronary artery disease: Novel insight from left atrial feature tracking
Source: Clin Cardiol. 2020 May 27;43(9):986–92. doi: 10.1002/clc.23395 (PMC7462186; doi:10.1002/clc.23395)
Supplement: Supplementary file 1 — Figure S1 Left atrial stiffness index is elevated in participants with ischemia and no obstructive coronary artery disease (INOCA, gold bar, n = 55) and heart failure with preserved ejection fraction (HFpEF, red bar, n = 15), compared to healthy reference controls (control, blue bar, n = 10). Left atrial stiffness index was estimated by dividing the ratio of early mitral inflow velocity‐to‐early diastolic strain rate (ie, E/e′SR) by left atrial reservoir strain. Early myocardial diastolic strain rate was measured in both the circumferential (panel A) and longitudinal (panel B) directions, by feature tracking of cine images, as described in detail in the body of the manuscript. Mitral inflow velocities were acquired using through plane phase contrast imaging, with the short axis image prescribed at the level of the mitral value leaflet tips. Group comparisons were main using a one‐way analysis of variance, with LSD post hoc comparisons. * indicates P < .05. [file CLC-43-986-s001.docx]

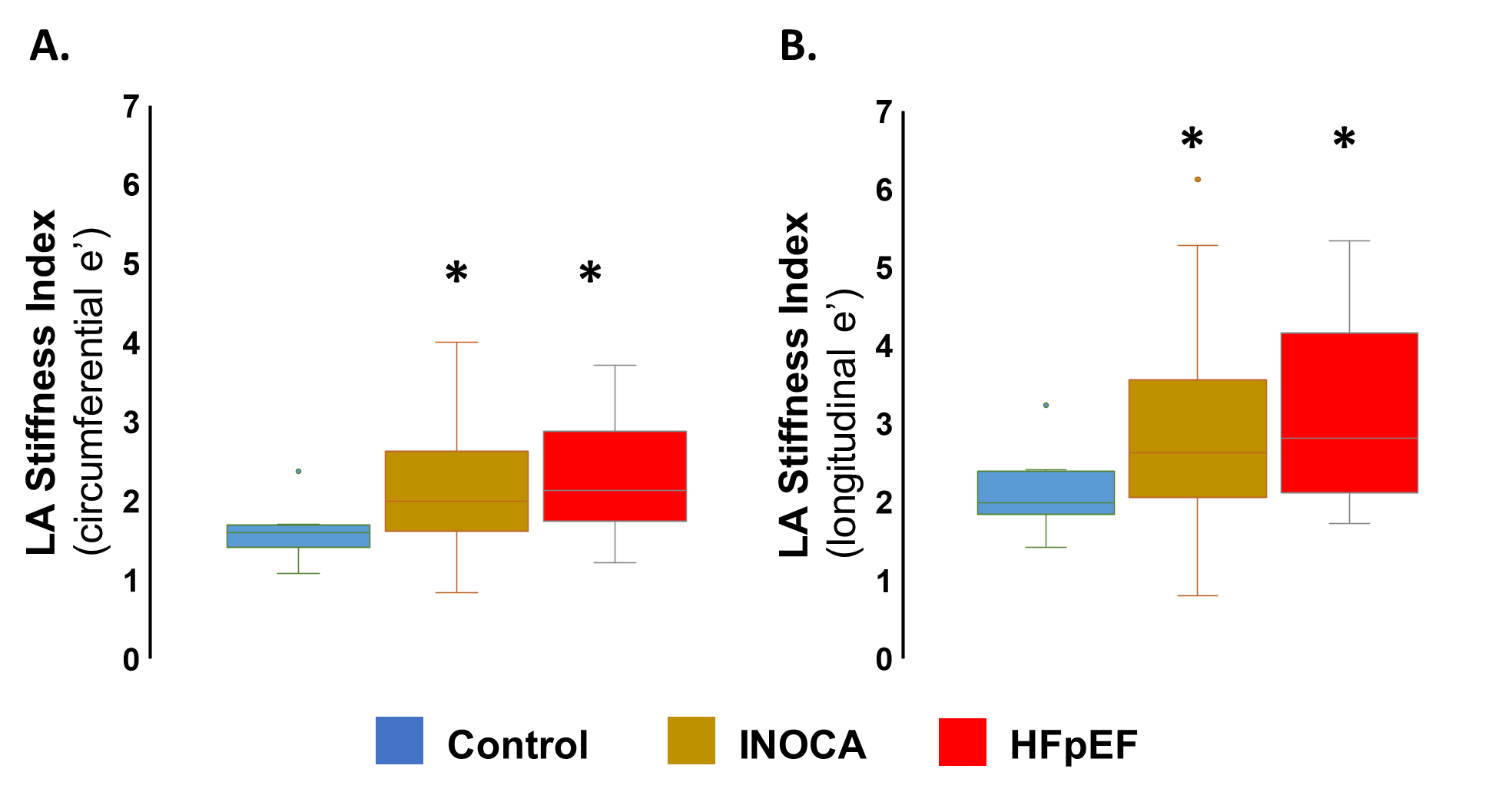


**Supplemental Figure 1.** Left atrial stiffness index is elevated in participants with ischemia and no obstructive coronary artery disease (INOCA, gold bar, n = 55) and heart failure with preserved ejection fraction (HFpEF, red bar, n = 15), compared to healthy reference controls (Control, blue bar, n = 10). Left atrial stiffness index was estimated by dividing the ratio of early mitral inflow velocity-to-early diastolic strain rate (i.e. E/e’_SR_) by left atrial reservoir strain. Early myocardial diastolic strain rate was measured in both the circumferential (Panel A) and longitudinal (Panel B) directions, by feature tracking of cine images, as described in detail in the body of the manuscript. Mitral inflow velocities were acquired using through plane phase contrast imaging, with the short axis image prescribed at the level of the mitral value leaflet tips. Group comparisons were main using a one-way analysis of variance, with LSD post hoc comparisons. * indicates P < 0.05.
